# Knockout of ABC transporters by CRISPR/Cas9 contributes to reliable and accurate transporter substrate identification for drug discovery

**DOI:** 10.3389/fphar.2022.1015940

**Published:** 2022-10-28

**Authors:** Dongyan Feng, Guorui Zhong, Qingxia Zuo, Yanbin Wan, Wanqing Xu, Changsheng He, Cailing Lin, Dongchao Huang, Feng Chen, Lizhen Huang

**Affiliations:** ^1^ School of Biology and Biological Engineering, South China University of Technology, Guangzhou, China; ^2^ Bioinformatics Institute, Agency for Science, Technology, and Research (A*STAR), Singapore, Singapore

**Keywords:** transporter substrate identification, ABC efflux transporters, CRISPR/Cas9, gene knockout, Caco-2

## Abstract

It is essential to explore the relationship between drugs and transporters in the process of drug development. Strong background signals in nonhuman MDCK or LLC-PK1 cells and overlapping interference of inhibitors or RNAi in human Caco-2 cells mean that an ideal alternative could be to knock out specific transporter genes in Caco-2 cells. However, the application of gene knockout (KO) to Caco-2 cells is challenging because it is still inefficient to obtain rapidly growing Caco-2 subclones with double-allele KO through long-term monoclonal cultivation. Herein, CRISPR/Cas9, a low cost but more efficient and precise gene editing technology, was utilized to singly or doubly knockout the P-gp, BCRP, and MRP2 genes in Caco-2 cells. By combining this with single cell expansion, rapidly growing transporter-deficient subclones were successfully screened and established. Bidirectional transport assays with probe substrates and three protease inhibitors indicated that more reliable and detailed data could be drawn easily with these KO Caco-2 models. The six robust KO Caco-2 subclones could contribute to efficient *in vitro* drug transport research.

## 1 Introduction

Drug transporters are recognized as key players in the processes of drug absorption, disposition, and elimination (Konig et al., 2013; Sorf et al., 2022), making it essential to explore the relationship between drug and transporters in the process of drug development. Members of the ATP-binding cassette (ABC) efflux transporter superfamily, including P-glycoprotein (P-gp), multidrug resistance-associated protein 2 (MRP2), and breast cancer resistance protein (BCRP), are the major drug efflux transporters that play important roles in drug bioavailability and disposition (Elliott et al., 2004; Griffin et al., 2011; Gupta et al., 2020; Martinec et al., 2021; Kikuchi et al., 2022).

As recommended by the FDA, canine MDCK, pig LLC-PK1, and human Caco-2 cells have been extensively used by researchers and pharmaceutical companies for decades as *in vitro* models for early estimates of drug absorption and efflux (FDA, 2020; Ding et al., 2021; Li et al., 2021; Huliciak et al., 2022; Mettler et al., 2022; Wang et al., 2022). Because of a lack of human transporters in MDCK and LLC-PK1, human transporter genes are usually introduced into cells transiently or stably to explore drug transport. However, overexpression MDCK or LLC-PK1 cells always have a strong background signal from the endogenous nonhuman transporters (Miyamoto et al., 2019; Crowe, 2021). This interferes with permeability and transporter studies, leading to less reliable data. The human epithelial cell line Caco-2 is originally derived from colon carcinoma cells. It can spontaneously differentiate into a polarized monolayer of cells with apical microvilli and intercellular tight junctions and express many transport systems, as found in the small intestine (Verhoeckx et al., 2015). Inactivation of specific transporters by inhibitors or RNAi has been frequently used in drug transport research with Caco-2 cells. However, many studies have found that the overlap of inhibitors with different transporters compromises the specificity of the inhibitors (Matsson et al., 2009; Mease et al., 2012). RNAi knockdown models are also faced with a specificity problem, because off-target effects are almost inevitable with RNAi (Neumeier and Meister, 2020).

To resolve the low specificity of transporter inhibitors or RNAi and achieve the accurate interpretation of specific drug–transporter interactions, an ideal alternative could be to target and knockout the specific transporter gene in cells. Zinc finger nucleases (ZFNs), the first generation of gene-editing technology, have been applied to knock out transporter genes in Caco-2 cells (Sampson et al., 2015). However, the application of gene editing to Caco-2 cells could be challenging because it is still inefficient to obtain rapidly growing Caco-2 subclones with double-allele knockout through long-term monoclonal cultivation. Several ZFN-mediated knockout Caco-2 cell lines are available, but are expensive. Pilot cell cloning experiments indicated that cell proliferation may even differ between different cell clones, which could influence the formation of monolayer cells in transporter studies (Crowe, 2021). This means that genomically-modified subclones need much more verification, including the monolayer formation rate. Few gene-modified Caco-2 lines with rapid growth potential have been developed after extensive screening and validation, highlighting the difficulties of applying gene editing in Caco-2 cells.

In the present study, CRISPR/Cas9, a low cost but more efficient and precise gene editing technology, was used to singly or doubly knockout the genes encoding P-gp, BCRP, or MRP2 in Caco-2 cells. By combining this with single cell expansion, rapidly growing transporter-deficient subclones were screened and established successfully. Bidirectional transport assays with a specific probe substrate and three protease inhibitors (PIs) were conducted to verify the performance of these knockout Caco-2 subclones. These rapidly growing gene knockout Caco-2 subclones could be useful *in vitro* models for drug transport research.

## 2 Materials and methods

### 2.1 Reagents and chemicals

5-(and-6)-carboxy-2′,7′-dichlorofluorescein (CDCF) and 5-(and-6)-carboxy-2′,7′-dichlorofluorescein diacetate (CDCFDA) were purchased from AAT Bioquest (Sunnyvale, CA, United States). Digoxin, estrone 3-sulfate (E3S), and lopinavir were purchased from Sigma-Aldrich (St. Louis, MO, United States). Nelfinavir was obtained from APExBIO Technology (Houston, TX, United States). Darunavir was purchased from Toronto Research Chemicals (North York, ON, Canada). A rabbit monoclonal P-gp antibody (ab120904), a rabbit monoclonal MRP2 antibody (ab172630) and a rabbit monoclonal GAPDH antibody (ab181602) were purchased from Abcam (Cambridge, MA, United States), and a rabbit monoclonal BCRP antibody (CST42078) was obtained from Cell Signaling Technology (Danvers, MA, United States). All other chemicals and solvents were of the highest grade commercially available.

### 2.2 Design and construction of sgRNA targeting the coding sequences of the P-gp, BCRP, and MRP2 genes

**FIGURE 1:**
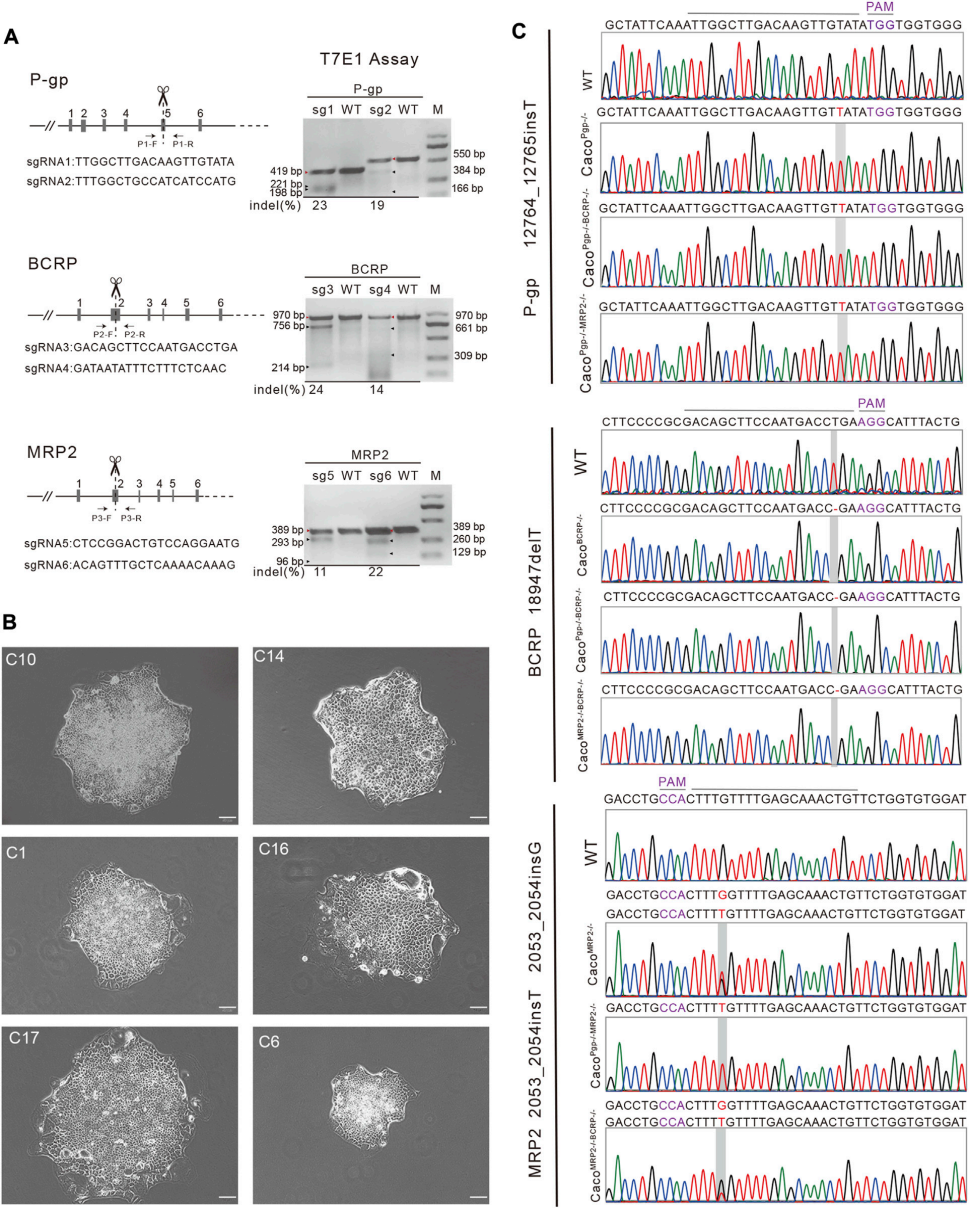
Establishment of single and double transporter KO cell lines clones. **(A)** Target sgRNA design for P-gp, BCRP and MRP2 and T7E1 assay. M: DNA marker. The red triangles are the parental bands and the black triangles are cleavage bands. **(B)** A portion of the harvested single colonies. Scale bar, 50 μm. **(C)** Sequencing results of the targeted allele in P-gp, BCRP and MRP2 knockout Caco-2 cell lines. PAM: protospacer adjacent motif.

The pCMV-Cas9 vector, which has a neomycin resistant gene for selection, was obtained from Addgene (41815). To generate single and double transporter KO cell lines, sgRNAs were designed to target exon 5 of the P-gp gene, exon 2 of the BCRP gene, and exon 2 of the MRP2 gene, based on the GN19 NGG rule using the CRISPR design tool (http://crispr.mit.edu). Additionally, Lindel, FORECasT and inDelphi were used to predict the Cas9 mediated gene editing results based on their databases (Allen et al., 2018; Shen et al., 2018; Chen et al., 2019). The sgRNA expression vectors were constructed as previously described (Mali et al., 2013; Zhong et al., 2018). In brief, oligonucleotides were annealed to form double-strand DNA and cloned into BbsI restriction enzyme-digested pU6-sgRNA cloning vectors. The sequences of the oligonucleotides were as follows: sgRNA1 (sg1), 5′-cac​cTT​GGC​TTG​ACA​AGT​TGT​ATA-3′ and 5′-aaa​cTA​TAC​AAC​TTG​TCA​AGC​CAA-3′; sgRNA2 (sg2), 5′-cac​cTT​TGG​CTG​CCA​TCA​TCC​ATG-3′ and 5′-aaa​cCA​TGG​ATG​ATG​GCA​GCC​AAA-3′; sgRNA3 (sg3), 5′-cac​cGA​CAG​CTT​CCA​ATG​ACC​TGA-3′ and 5′-aaa​cTC​AGG​TCA​TTG​GAA​GCT​GTC-3′; sgRNA4 (sg4), 5′-cac​cGA​TAA​TAT​TTC​TTT​CTC​AAC-3′ and 5′-aaa​cGT​TGA​GAA​AGA​AAT​ATT​ATC-3′; sgRNA5 (sg5), 5′-cac​cCT​CCG​GAC​TGT​CCA​GGA​ATG-3′ and 5′-aaa​cCA​TTC​CTG​GAC​AGT​CCG​GAG-3′; and sgRNA6 (sg6), 5′-cac​cAC​AGT​TTG​CTC​AAA​ACA​AAG-3′ and 5′-aaa​cCT​TTG​TTT​TGA​GCA​AAC​TGT-3′ (Figure 1A). All the constructs were confirmed by sequencing (BGI, Guangzhou, China). The plasmids were purified using the Endo-free Plasmid Mini kit (Omega Bio-Tek, Norcross, GA, United States).

### 2.3 Cell culture and transfection

HEK 293T cells were obtained from Cell Bank/Stem Cell Bank, Chinese Academy of Sciences and cultured in Dulbecco’s Modified Eagle’s Medium (DMEM) supplemented with 10% fetal bovine serum (FBS; Life Technologies), 100 U/mL penicillin, and 100 μg/mL streptomycin at 37°C with 5% CO_2_ incubation. Caco-2 cells were obtained from the Shanghai Institute of Cell Biology, the Chinese Academy of Sciences (Shanghai, China) and cultured in MEM medium with 10% FBS, 1% (v/v) minimum Eagle’s medium nonessential amino acids, 2 mM L-glutamine, 1 mM sodium pyruvate, 100 U/mL penicillin, and 100 μg/mL streptomycin at 37°C with 5% CO_2_. The culture medium was replaced every second day.

1.0 × 10^6^ cells were cultured in 6-well plates 24 h prior to transfection and reached 70%–80% confluence at time of transfection. Cells were co-transfected with 0.5 µg of pCMV-Cas9 and 0.5 µg of pU6-sgRNA plasmid using GenJet™ *In Vitro* DNA Transfection Reagent (SignaGen, Rockville, MD) following the manufacturer’s recommendations.

### 2.4 T7E1 nuclease assay for genome modification

T7 Endonuclease I (T7E1), which recognizes and cleaves non-perfectly matched DNA, cruciform DNA structures, Holliday structures or junctions and heteroduplex DNA, was used to detect the gene editing efficiency. Forty-eight hours post-transfection, the HEK 293T cells were harvested and genomic DNA was extracted using a DNA Mini Prep kit (Omega). The target regions, exon 5 of the P-gp gene, exon 2 of the BCRP gene, and exon 2 of the MRP2 gene, were PCR amplified using specific primers (P1-F: 5ʹ-ggg​tgt​ctt​gga​cta​ggt​tgg​t-3ʹ, P1-R: 5ʹ-tat​ttc​tga​ctt​cac​agg​gct​ctc-3ʹ; P2-F: 5ʹ-agc​tgc​tca​ttg​ccg​cac​at-3ʹ, P2-R: 5ʹ-tgt​gaa​gcc​ttg​agc​aga​cga​g-3ʹ; P3-F: 5ʹ-tgt​gtg​aaa​gca​gtg​gga​tgt​g-3ʹ, P3-R: 5ʹ-tgg​ctc​tac​ctg​aga​caa​tgg​c-3ʹ). For the T7E1 assay, the PCR products were denatured and reannealed for heteroduplex formation. Next, 19 µl of annealed products were incubated with 1 µl T7E1 nuclease (New England Biolabs, MA, United States) at 37°C for 30 min, followed by analysis on 2% agarose gels. The indel percentage was determined by the formula:

indel(%)=100×[1−(1−(b+c)/(a+b+c))1/2]

where a is the integrated intensity of the undigested PCR product, and b and c are the integrated intensities of each cleavage product (Ran et al., 2013).

### 2.5 Generation of transporter KO Caco-2 cell clones

Two days after transfection, cells were cultured with selective medium (G418, 800 μg/ml) for 3–4 days. Then, the cells were trypsinized and seeded at low density (100 cells/10-cm dish) without G418 and allowed to expand and form discernible colonies for 2–3 weeks. The single-cell colonies were isolated using cloning rings and transferred to 24-well plates for monoclonal expansion. Once the 24-well culture plate clones expanded to high confluence, they were passaged to a 6-well culture plate. A small portion of the cells was assessed for knockout of the targeted gene. The genomic DNA was extracted by incubation in lysis buffer containing Triton X-100 (0.45%, Sigma) and proteinase K (1 mg/ml, Merck, Germany) at 37°C for 1 h, 56°C for 1 h, followed by 95°C for 10 min. The target regions from individual cell colonies were amplified by PCR using the previously described PCR primers (P1-F/P1-R, P2-F/P2-R, P3-F/P3-R) and the PCR product was sequenced by Sanger sequencing. DNA mutations were identified by sequence alignment between the sequenced allele and wild-type allele by SnapGene.

### 2.6 Western blot analysis

The cells were harvested and lysed in RIPA buffer (50 mM Tris-HCL pH 7.5, 150 mM NaCl, 1% IPEGAL, 0.5% deoxycholate, 5 mM EDTA). Cell proteins were quantified using a BCA Protein Assay Kit (Thermo Fisher Scientific, United States). For protein immunoblot analysis, proteins (50 µg/lane) were separated on 12% SDS-PAGE gel and transferred onto PVDF membranes (0.45 µm pore size, IPVH00010; Millipore, Billerica, MA, United States). Membranes were washed, blocked at room temperature in TBST containing 5% nonfat dry milk powder (Newprobe, China) for 1 h, and then incubated overnight with primary antibody at the appropriate dilution (P-gp, 1:2000; BCRP, 1:1000; MRP2, 1:3000; GAPDH, 1:5000) at 4°C. A secondary horseradish peroxidase-conjugated antibody (Abcam) was applied to allow a color reaction for chemiluminescence detection (SUPERSIGNAL® West Pico Chemiluminescent Substrate; Thermo Fisher Scientific, NH, United States).

### 2.7 TEER measurement and bidirectional transport assay

Caco-2 WT and KO cell lines were seeded onto polycarbonate membrane filters (1 µm pore size, 0.33 cm^2^ growth area) inside a 24-well hanging cell culture insert (Millicell, Germany) at a density of 4 × 10^4^ cells/well. The cell culture inserts were placed in 24-well tissue culture plates with 0.9 ml of medium outside (basolateral side) and 0.2 ml of medium inside (apical side). The medium was replaced every second day. Cells were cultured for 16–22 days to obtain differentiated monolayers with tight junctions and polarized transporter expression. The integrity of the monolayers was monitored by measuring transepithelial electrical resistance (TEER) every 2 days before the bidirectional transport experiments using a Millicell-ERS epithelial voltmeter (Millipore Corporation, United States). The transporter experiment was only conducted on cell monolayers with TEER values above 300 Ω·cm^2^.

First, the probe substrates 5 μM digoxin, 5 μM E3S, and 10 μM CDCF (added as CDCFDA) were used to validate the functional activity of P-gp, BCRP, and MRP2, respectively (Sampson et al., 2015). Then, we conducted in Caco-2 WT and KO cell lines to investigate the apparent permeability coefficients (Papp) and efflux ratio (ER) of 5 μM nelfinavir, 5 μM lopinavir, and 5 μM darunavir. All these studies were conducted by following similar procedures. Briefly, on the day of the experiment, the culture medium was aspirated. The cell monolayers were twice washed with HBSS (PH 7.4) at 37°C and then incubated with HBSS at 37°C for 30–60 min. Then, HBSS was aspirated from both sides. Test chemicals were diluted from 10 mM DMSO stock solutions to 5 or 10 µM in HBSS. For A-to-B transport, 0.2 ml of compound-containing solution was loaded into the apical side (AP) and 0.9 ml of HBSS buffer was loaded into the basolateral side (BL). For B-to-A transport, 0.2 ml of HBSS buffer was loaded into the apical side and 0.9 ml of compound-containing solution was loaded into the basolateral side. After incubation at 37°C for 1 h, samples were immediately combined with an equal volume of acetonitrile:H_2_O (85:15) and stored at 4°C until sample analysis.

For MRP2, the nonfluorescent compound CDCFDA was used as the probe substrate. This compound passively diffuses into cells and is hydrolyzed by intracellular esterases to the fluorescent product, CDCF, which is then rapidly excreted by MRP2 (Sampson et al., 2015). After treatment, samples were transferred to black-walled 96-well assay plates. A standard curve of 2-fold serial dilution from 10 µM CDCF was generated. The CDCF concentration was measured based on the fluorescence in a microplate fluorescence reader (Tecan, Switzerland) set at 485 nm and 530 nm for the excitation and emission wavelengths, respectively.

### 2.8 LC-MS/MS analysis

The concentrations of the test chemicals in the samples were analyzed by liquid chromatography-tandem mass spectrometry (LC-MS/MS) using a Shiseido NANOSPACE 1312 HPLC system (Tokyo, Japan) coupled with an AB Sciex 4000 Q TRAP™ (Ontario, Canada). Samples (20 µl) were injected onto a Venusil MP C18 column (50 × 2.1 mm, 5 μm, Tianjin, China) and eluted by a mobile phase gradient optimized for each test chemical (mobile phase A was composed of acetonitrile and water at a ratio of 5:95 (v/v) and B was acetonitrile and water, 95:5 (v/v)). The flow rate was 0.3 mL/min. Analytes were quantitated using multiple reaction monitoring specific for each analyte and internal standard parent–product ion pairs. The precursor and the product ions generated were 798.6/651.5 for digoxin, 349.2/269.3 for E3S, 568.4/330.2 for nelfinavir, 629.3/155.1 for lopinavir, 548.2/392.1 for darunavir. The peak areas of analytes and internal standards and the resulting ratios were quantified using Analyst 1.5 (AB SCIEX, CA, United States).

### 2.9 Calculations

The apparent permeability coefficients (Papp) across cell monolayers in both the AP-to-BL (Papp (A-to-B) and BL-to-AP (Papp (B-to-A) directions were calculated as follows:

$$P_{app}=\frac{dQ/dt}{A\times C_{0}}$$

where A is the surface area of the transwell filter, C_0_ is the initial drug concentration in the donor side, dQ is the amount of transported drug, and dt is time elapsed. The Efflux Ratio was calculated according to the equation:

$$ER=\frac{P_{app}\left(B-to-A\right)}{P_{app}\left(A-to-B\right)}$$

According to FDA guidance, an ER ≥ 2 suggests active transport across the cell monolayer, identifying the compound as an apical efflux transporter substrate (FDA, 2020).

### 2.10 Statistical analysis

TEER measurement was analyzed by two-way analysis of variance (ANOVA) to determine the statistically significant differences. All transport assays were performed in three independent experiments and one-way analysis of variance (ANOVA) was used for multiple comparisons to determine the statistically significant differences. The results of the experiments were expressed as the mean ± S.D. Differences were regarded as significant when the *p* value was <0.05.

## 3 Results

### 3.1 Generation of efflux transporter knockout and rapidly growing Caco-2 subclones with CRISPR/Cas9 and single cell expansion

**FIGURE 2:**
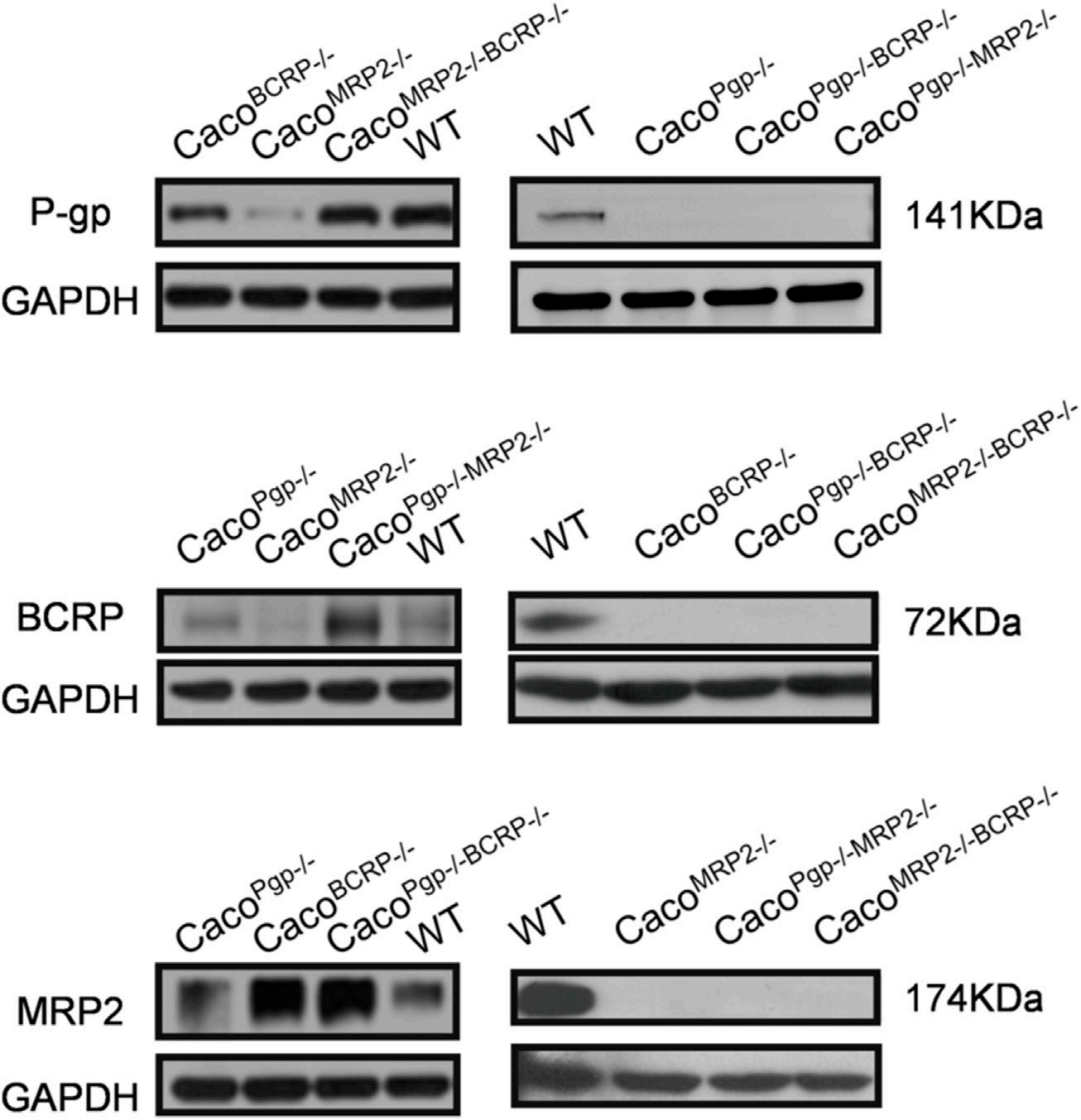
Western blot analysis of P-gp, BCRP and MRP2 protein expression in WT and KO cell lines. The left and right sides experiments were independent experiments.

To generate single and double transporter KO cell lines, sgRNAs were designed to target exon 5 of P-gp, exon 2 of BCRP, and exon 2 of MRP2 (Figure 1A). Two sgRNAs for each target were designed based on cleavage efficiency prediction and off-target analysis. The T7E1 assay indicated that the indel frequency of the constructed sgRNA was 11%–24% in HEK 293T cells (Figure 1A). Next, the sgRNAs with higher indel frequencies (such as sg1, sg3, and sg6) were selected and co-transfected with Cas9 into Caco-2 cells to generate Caco^Pgp−/−^, Caco^BCRP−/−^, and Caco^MRP2−/−^ single KO cell clones. We isolated rapidly growing single colonies from the transfected cells and analyzed the indel mutations in these isolated subclones (Figures 1B,C). In total, 9 Caco^Pgp−/−^ subclones, 10 Caco^BCRP−/−^ subclones, 9 Caco^MRP2−/−^ subclones, 8 Caco^Pgp−/−BCRP−/−^ subclones, 14 Caco^Pgp−/−MRP2−/−^ subclones and 9 Caco^MRP2−/−BCRP−/−^ subclones were obtained. According to the Sanger sequencing results, there is a T nucleotide insertion in g12764_12765 of the P-gp gene in Caco^Pgp−/−^-C10, a T nucleotide deletion in g18947 of the BCRP gene in Caco^BCRP−/−^-C14, and a T/G nucleotide insertion in g2053_2054 of each MRP2 gene allele in Caco^MRP2−/−^-C1 (Figure 1C). These P-gp, BCRP, and MRP2 single KO subclones contained out-of-frame indels resulting in the generation of a premature stop codon. Then the second round of CRISPR/Cas9 transfections and clone isolation was performed with these single KO clones to establish the double KO subclones. Caco^Pgp−/−BCRP−/−^-C16 and Caco^Pgp−/−MRP2−/−^-C17 were generated from clone Caco^Pgp−/−^-C10, and Caco^MRP2−/−BCRP−/−^-C6 was generated from clone Caco^MRP2−/−^-C1. Finally, the expression of the transporter protein in all 6 cell clones was determined by western blot analysis (Figure 2). A slight decrease of the expression of P-gp and BCRP was observed in Caco^MRP2−/−^, and a small increase of the expression of MRP2 was observed in Caco^BCRP−/−^ and Caco^Pgp−/−BCRP−/−^. For each transporter, it was completely absent in the corresponding single or double KO cell clones and present in WT and the rest of the KO clones (P-gp, 141 kDa; BCRP, 72 kDa; MRP2, 174 kDa). The above results confirmed the successful establishment of stable Caco-2 subclones with single and double transporter knockout.

**FIGURE 3:**
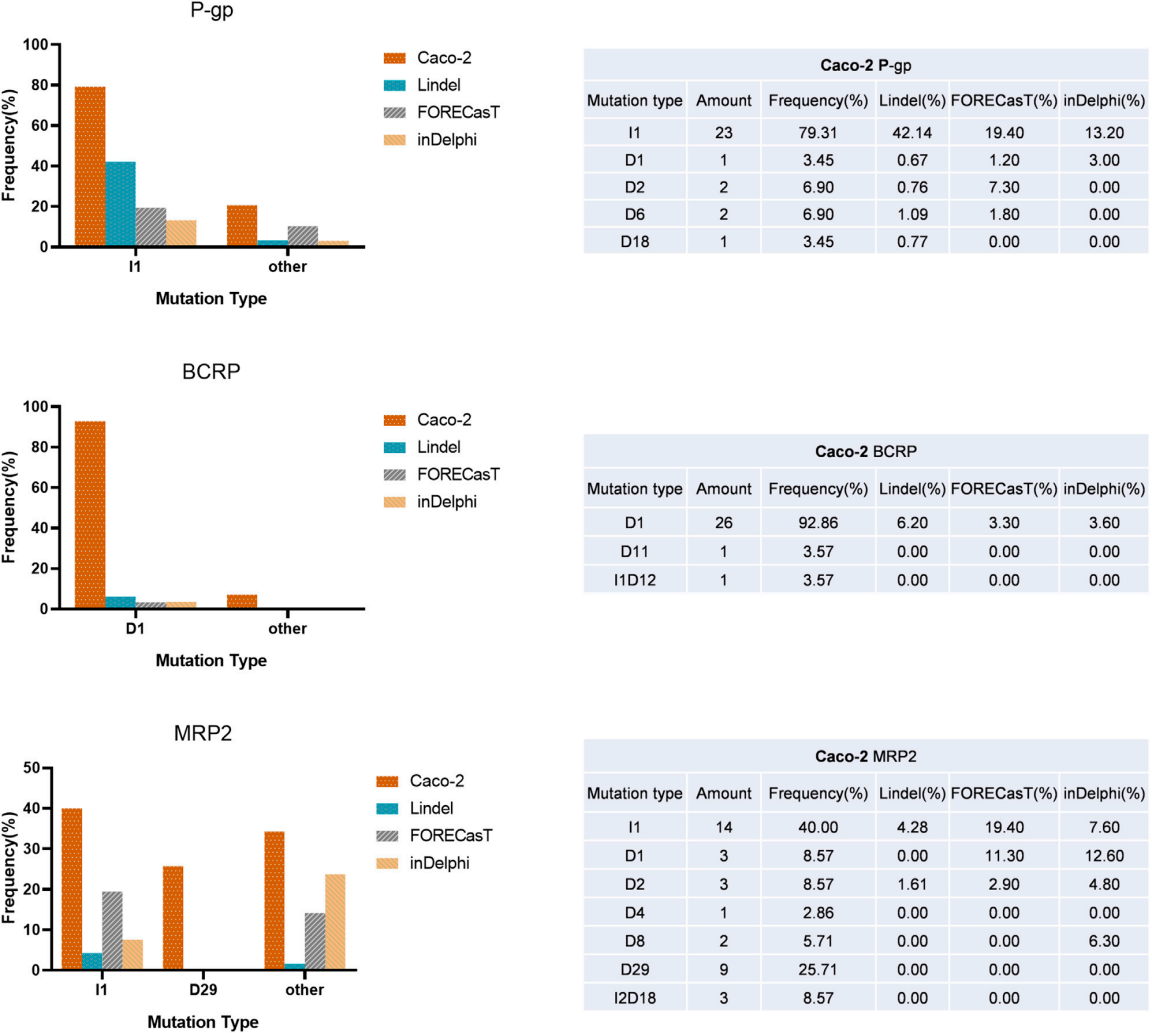
A summary of indel outcomes of KO clones and comparing the indel frequency with the three prediction models. Left: the frequency of indels. Right: the summary of mutation type for P-gp, BCRP and MRP2 targets. The abbreviation of mutation type: I, insertion; D, deletion; the number means the nucleotides number indel or insertion (e.g., D1 means 1 nucleotide deleted in target site.).

In addition, we noted a strong bias in indel type of the isolated subclones (Figure 3, Figure 1B), which made the repair outcomes of our two round knockouts almost the same (except for the very similar results for the MRP2 gene, the first-round repair outcome was heterozygous T/G nucleotide insertion in g2053_2054 of Caco^MRP2−/−^-C1, while the second-round repair outcome was homozygous T nucleotide insertion in g2053_2054 of Caco^Pgp−/−MRP2−/−^-C17). When comparing the indel outcomes of our results with the frequently used indel prediction models (Lindel, FORECasT and inDelphi), we found that although the most frequent indel was not always the same as that predicted by the models, all the prediction models covered most of the DSB repair outcomes in Caco-2 cells. Thus, the indel prediction models could provide a valuable reference for indel types but may be less accurate for indel frequency prediction in Caco-2 cells.

### 3.2 TEER changes of transport KO Caco-2 subclones during monolayer formation

**FIGURE 4:**
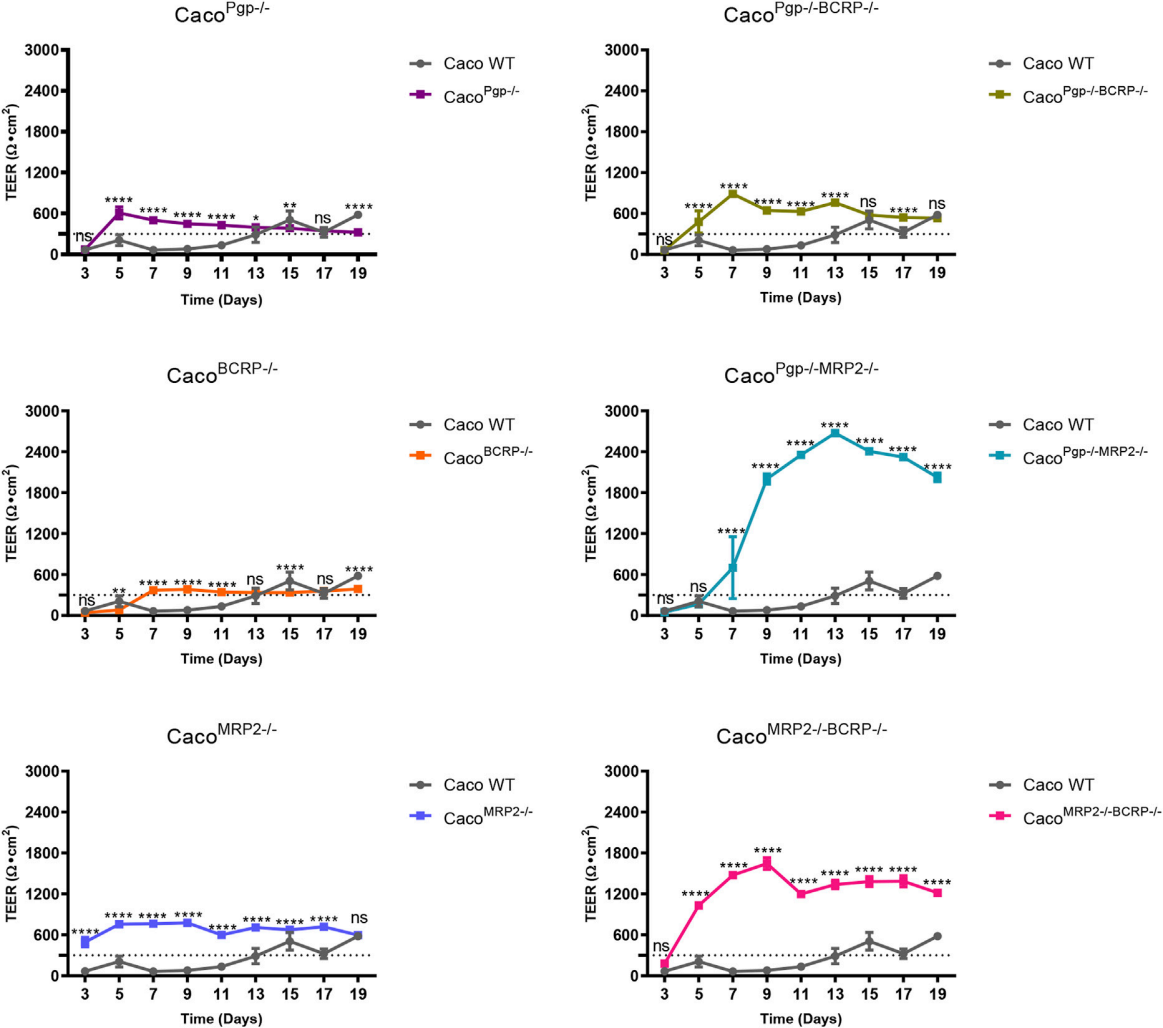
The TEER changes of each KO clone compared with the WT cell. Each bar represents the mean ± SD.; *n* = 5. The dotted line indicates 300 Ω·cm^2^. ns: not significant; *p < 0.05; **p < 0.01; ***p < 0.001; ****p < 0.0001.

To perform the transport assay, the cells need to be seeded on a transwell plate to differentiate into a monolayer with polarized transport protein expression. The TEER of KO and WT cells were routinely measured every 2 days from day 3 to day 19 during monolayer development. As shown in Figure 4, they were all above 300 Ω·cm^2^ by 19 days before the start of functional transport assays, but all the selected KO clones grew rapidly and showed fast-rising TEER values compared with WT. The TEER of the KO clones reached 300 Ω·cm^2^ by 5–7 days, while WT cells needed 13 days, and the average TEER values of the KO clones were also larger than the WT cells. Such differences were extremely apparent in the MRP2 KO clones, where the highest TEER reached 2600 Ω·cm^2^ by 13 days and 1,600 Ω·cm^2^ by 9 days in Caco^Pgp−/−MRP2−/−^ and Caco^MRP2−/−BCRP−/−^ KO cells, respectively (Figure 4). In WT cells, the TEER reached a peak of 580 Ω·cm^2^ by 19 days.

### 3.3 Bidirectional transport assays of Caco-2 KO subclones with probe substrates

**FIGURE 5:**
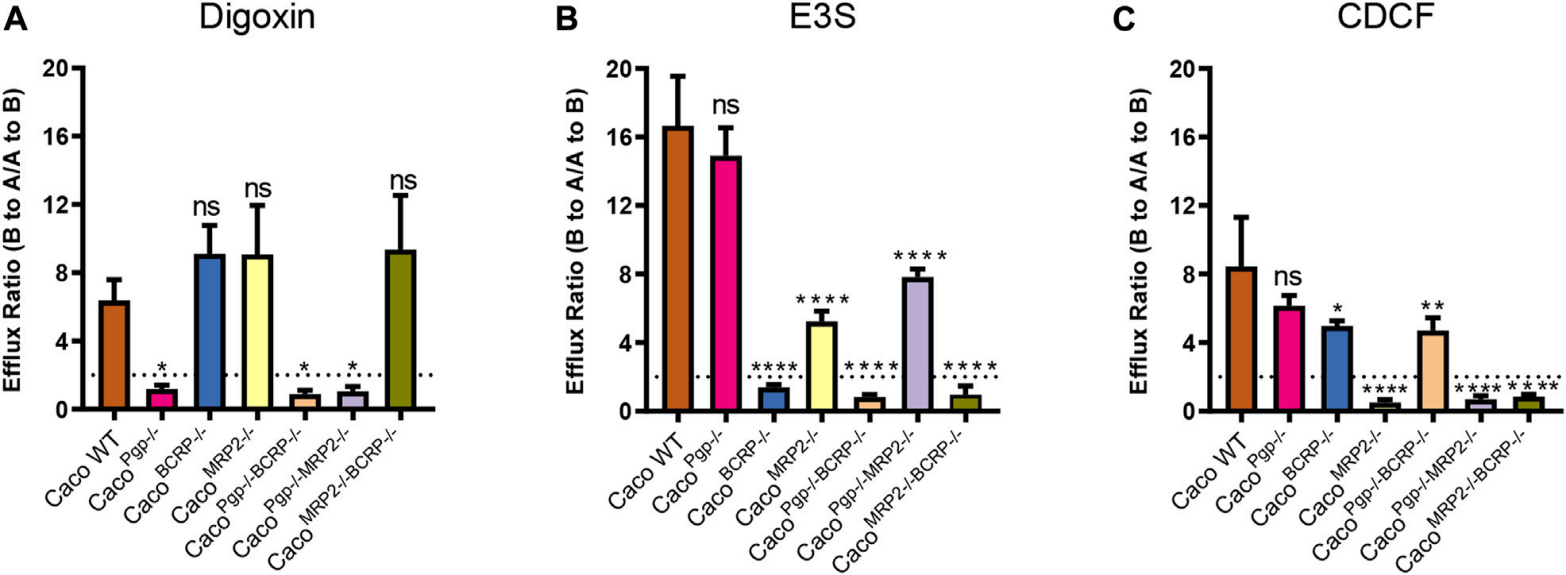
Efflux ratios for probe substrates digoxin **(A)**, estrone 3-sulfate **(B)**, and CDCF **(C)** in WT and KO cell lines. Each bar represents the mean ± SD.; n = 3. The dotted line indicates the ER = 2. ns: not significant; *p < 0.05; **p < 0.01; ***p < 0.001; ****p < 0.0001.

The efflux functions of P-gp, BCRP, and MRP2 in the KO cell lines were further evaluated. Digoxin, E3S, and CDCF were chosen as model substrates for P-gp, BCRP, and MRP2, respectively. The A-to-B and B-to-A permeability values and the resultant efflux ratios are shown in Figure 5 and Table 1. The efflux ratio of the P-gp substrate digoxin was 6.4 in WT cells and decreased to 1 in the P-gp single- and double-KO cells. The BCRP ubstrate, E3S, exhibited a high efflux ratio of 16.6 in the WT cells, which was reduced to 0.8–1.3 in the BCRP single- and double-KO cell lines. Also, as the MRP2 substrate (Kopplow et al., 2005), the efflux ratio of E3S was reduced significantly in MRP2 single- and double-KO cell lines compared with WT. The efflux ratio of CDCF, a MRP2 substrate, was reduced from 8.4 in WT cells to ˜1 in the MRP2 single- and double-KO cell lines. Slight reductions of the CDCF efflux ratio were also observed in BCRP single- and double-KO cells. These results indicate that the single- and double-KO cell lines completely lost the transporter function.

**TABLE 1:** Functional activity of efflux transporters in KO cells.

Substrate/Transporter	Cell line	Papp (×10^−6^cm/s)		Efflux ratio
		A-to-B	B-to-A	
Digoxin/P-gp	WT	1.48 ± 0.21	9.33 ± 1.34	6.38 ± 1.21
	Caco^Pgp−/−^	1.42 ±0.20	1.67 ±0.23	1.20 ±0.21
	Caco^BCRP−/−^	1.66 ± 0.42	14.78 ± 1.85	9.11 ± 1.66
	Caco^MRP2−/−^	2.05 ± 0.51	17.61 ± 1.13	9.08 ± 2.87
	Caco^Pgp−/−BCRP−/−^	2.42 ± 0.64	2.08 ± 0.12	0.90 ± 0.21
	Caco^Pgp−/−MRP2−/−^	1.95 ±0.92	1.87 ±0.40	1.05 ±0.29
	Caco^MRP2−/−BCRP−/−^	2.03 ± 0.55	17.88 ± 0.63	9.36 ± 3.17
Estrone 3-Sulfate/BCRP	WT	0.67 ± 0.12	10.87 ± 0.20	16.65 ± 2.90
	Caco^Pgp−/−^	1.53 ± 0.24	22.48 ± 1.01	14.90 ± 1.63
	Caco^BCRP−/−^	1.15 ± 0.11	1.58 ± 0.08	1.36 ± 0.18
	Caco^MRP2−/−^	2.91 ± 0.32	15.32 ± 3.10	5.24 ± 0.59
	Caco^Pgp−/−BCRP−/−^	1.88 ± 0.02	1.54 ± 0.30	0.820 ± 0.15
	Caco^Pgp−/−MRP2−/−^	3.23 ± 0.08	25.34 ± 2.06	7.830 ± 0.46
	Caco^MRP2−/−BCRP−/−^	0.97 ± 0.04	0.92 ± 0.49	0.96 ± 0.51
CDCF/MRP2	WT	1.05 ± 0.34	8.25 ± 0.63	8.45 ± 2.86
	Caco^Pgp−/−^	1.94 ± 0.10	11.95 ± 1.61	6.16 ± 0.59
	Caco^BCRP−/−^	2.14 ± 0.08	10.63 ± 0.96	4.97 ± 0.30
	Caco^MRP2−/−^	2.33 ± 0.62	1.10 ± 0.13	0.50 ± 0.17
	Caco^Pgp−/−BCRP−/−^	2.02 ± 0.20	9.42 ± 0.55	4.81 ± 0.75
	Caco^Pgp−/−MRP2−/−^	2.41 ± 0.55	1.64 ± 0.46	0.69 ± 0.21
	Caco^MRP2−/−BCRP−/−^	3.48 ± 0.51	3.05 ± 0.80	0.87 ± 0.12

A-to-B, apical side to basolateral side transport; B-to-A, basolateral side to apical side transport; n = 3

### 3.4 Interactions of PIs with P-gp, BCRP, and MRP2 efflux transporters in Caco-2 cells

**FIGURE 6:**
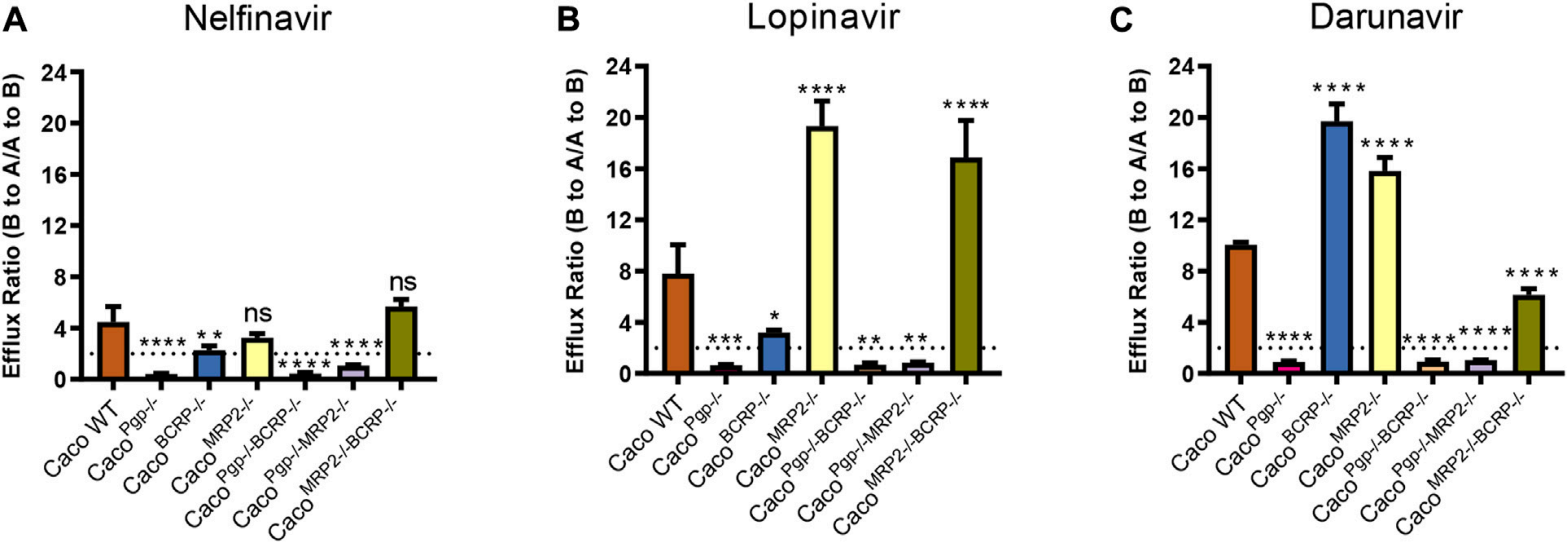
Efflux ratios for 5 μM nelfinavir **(A)**, 5 μM lopinavir **(B)** and 5 μM darunavir **(C)** in WT and KO cell lines. Data represents the mean ± SD; *n* = 3. The dotted line indicates the ER = 2. ns: not significant; *p < 0.05, **p < 0.01, ***p < 0.001, ****p < 0.0001.

To further test the utility of our transporter KO model, bidirectional transport assays were conducted to determine whether nelfinavir, lopinavir, and darunavir, protease inhibitors (PIs) in HIV treatment, are substrates of those efflux transporters. The Papp and ER of nelfinavir, lopinavir, and darunavir tested in this study are shown in Figure 6 and Table 2.

**TABLE 2:** Permeability and efflux ratios of nelfinavir (5 μM), lopinavir (5 μM) and darunavir (5 μM) across cell monolayers.

PIs	Cell line	Papp (×10^−6^cm/s)		Efflux ratio
		A-to-B	B-to-A	
Nelfinavir	WT	2.78 ± 0.39	12.18 ± 1.86	4.47 ± 1.19
	Caco^Pgp−/−^	4.17 ±0.17	2.18 ±0.43	0.41 ±0.04
	Caco^BCRP−/−^	2.21 ±0.41	4.88 ±0.17	2.25 ±0.34
	Caco^MRP2−/−^	4.53 ±0.26	14.67 ±0.99	3.25 ± 0.31
	Caco^Pgp−/−BCRP−/−^	7.54 ±0.24	3.22 ±0.62	0.43 ±0.09
	Caco^Pgp−/−MRP2−/−^	4.23 ± 0.22	4.51 ± 0.63	1.07 ± 0.42
	Caco^MRP2−/−BCRP−/−^	2.79 ± 0.28	17.89 ± 1.89	6.41 ± 0.35
Lopinavir	WT	3.25 ± 0.59	24.49 ± 2.04	7.81 ± 2.24
	Caco^Pgp−/−^	10.21 ± 0.27	6.59 ± 0.62	0.65 ± 0.05
	Caco^BCRP−/−^	5.79 ± 0.64	18.45 ± 0.98	3.20 ± 0.19
	Caco^MRP2−/−^	1.04 ± 0.04	20.05 ± 2.26	19.32 ± 1.95
	Caco^Pgp−/−BCRP−/−^	15.22 ± 1.44	10.26 ± 0.82	0.68 ± 0.12
	Caco^Pgp−/−MRP2−/−^	13.77 ± 1.01	11.54 ± 0.96	0.84 ± 0.07
	Caco^MRP2−/−BCRP−/−^	1.13 ± 0.03	19.04 ± 3.05	16.87 ± 2.88
Darunavir	WT	2.65 ± 0.22	26.59 ± 2.06	10.06 ± 0.19
	Caco^Pgp−/−^	15.09 ± 0.68	13.58 ± 0.80	0.90 ± 0.07
	Caco^BCRP−/−^	1.75 ± 0.13	34.320 ± 0.19	19.70 ± 1.350
	Caco^MRP2−/−^	1.90 ± 0.14	29.84 ± 0.24	15.81 ± 1.06
	Caco^Pgp−/−BCRP−/−^	12.59 ± 1.31	11.54 ± 0.38	0.93 ± 0.13
	Caco^Pgp−/−MRP2−/−^	10.06 ± 0.44	10.42 ± 0.20	1.04 ± 0.03
	Caco^MRP2−/−BCRP−/−^	4.43 ± 0.63	26.97 ± 0.79	6.13 ± 0.49

A-to-B, apical side to basolateral side transport; B-to-A, basolateral side to apical side transport; n = 3.

In WT cells, the ERs of the three PIs were all around 4–10, indicating that they could be transported by apical efflux transporters. The efflux ratio of nelfinavir dropped to˜1 or even lower in P-gp single- and double-KO cells and to˜2 in BCRP single-KO cells. Although the BCRP transporter was still expressed in Caco^Pgp−/−MRP2−/−^ cells, no active transport was observed (Figure 6A). In contrast, the ERs of the rest of the KO cells did not differ significantly from WT. The above results suggested that P-gp was the main transporter for nelfinavir and BCRP had little contribution to its transport.

For lopinavir, in all the P-gp KO cells, the efflux ratios were <1 (0.6–0.8). Similar to nelfinavir, the efflux ratio of lopinavir was also slightly reduced (from 7.8 to 3.2) in the Caco^BCRP−/−^ cell line (Figure 6B). Interestingly, the Papp (A-to-B) values were reduced to ˜1 × 10^–6^ cm/s in Caco^MRP2−/−^ cells and Caco^MRP2−/−BCRP−/−^ cells, while the values for Papp (B-to-A) were not significantly changed (Table 2), which resulted in higher efflux ratio of lopinavir in Caco^MRP2−/−^ cells and Caco^MRP2−/−BCRP−/−^ cells than in WT cells. These results indicate that lopinavir is a main substrate of P-gp, but BCRP also has an impact on its transport.

The ER of darunavir was significantly decreased (0.9–1.0) in the P-gp KO cell lines (Figure 6C). The ER in Caco^MRP2−/−BCRP−/−^ was reduced significantly for the increase of Papp (A-to-B) permeability (from 2.6 × 10^–6^ to 4.4 × 10^–6^ cm/s) compared with WT cells (Table 2). Furthermore, the ERs of darunavir in Caco^BCRP−/−^ and Caco^MRP2−/−^ cells were higher than those in WT cells because of the decrease of Papp (A-to-B) permeability (from 2.6 × 10^–6^ to 1.7–1.8 × 10^–6^ cm/s) and the increase in Papp (B-to-A) permeability (from 26.6 × 10^–6^ to 29.8–34.3 × 10^–6^ cm/s). The above results clearly demonstrate that darunavir is a substrate for P-gp only.

## 4 Discussion

Robust cell models have been considered to be absolutely critical for *in vitro* measurement of the cellular transport of drugs and food components (Verhoeckx et al., 2015). Here, we successfully established six rapidly growing Caco-2 subclones with P-gp, BCRP, and MRP2 single or double knockout by combining CRISPR/Cas9 technology with single cell expansion.

Because of the high gene editing efficiency and low off-target effects of the CRISPR/Cas9 technology, many more Caco-2 subclones with double-allele knockout were obtained by one or two rounds of gene editing. Although three prediction models (Lindel, FORECasT and inDelphi) covered most of the gene editing outcomes in Caco-2 cells, the most frequent indel in Caco-2 cells was not the same as in three models. Indel prediction models provided less accurate predictions for indel frequency in Caco-2 cells. Most of Caco-2 subclones grew rapidly even through the long-term single cell expansion. The expression of untargeted transporter proteins might be affected in some knockout cells, which might be due to the adaption or compensation by the relevant genes when the target gene was knocked out. However, our data showed that the transporting capability remained nearly the same and the expression changes did not seem to affect ER apparently.

During the monolayer culture in transwell plates, the TEER values of the six isolated KO subclones ascended faster and finally higher than the wild-type Caco-2 cells. More than 300 Ω·cm^2^ were observed in most subclones within as little as 5 days post-seeding, even more than 1,000 Ω·cm^2^ at day 7 in the Caco^Pgp−/−MRP2−/−^ and Caco^MRP2−/−BCRP−/−^ KO subclones. This implied that better and faster tight junction formation occurred in those rapidly growing subclones. Crowe et al. (Crowe, 2021) demonstrated that the rapidly growing subclones acquired through single cell expansion worked well at day 6 post-seeding when the TEER value exceeded 1,000 Ω·cm^2^. Therefore, here the fast-growing KO Caco-2 subclones could also be rapid models, and would be a welcome addition to the testing regime of new chemical entities.

The bidirectional transport studies with probe substrate clearly indicated that the single- and double-KO subclones completely lost the function of the targeted knockout transporter but retained the function of the nontarget transporters. Furthermore, clearer data can be obtained from KO cells compared with probe substrate studies in Caco-2 using inhibitors. When employing the inhibitor, 1 µM fumitremorgin C (FTC, BCRP inhibitor) selectively inhibited the BCRP transporter, whereas 10 µM FTC only partially inhibited P-gp (Mease et al., 2012). However, it was obvious that the probe substrate E3S only failed to be transported within all the BCRP-knockout cells, showing no interference among different knockout cells. In addition, a weak decrease of efflux was also observed in MRP2-knockout cells, further indicating that E3S was partially transported by MRP2 (Kopplow et al., 2005; Volpe, 2016). The efflux comparison of the same probe substrate with KO cells and inhibitor-treated cells also showed that the KO cells would contribute to more reliable and detailed data for efflux study.

The panel of six KO Caco-2 subclones could also be successfully applied in studying the efflux of three PIs (nelfinavir, lopinavir, and darunavir). The bidirectional transport assays confirmed that P-gp played a key role in transport of the three PIs, just as reported previously with *in vivo* and *in vitro* models (Agarwal et al., 2007; Kaddoumi et al., 2007; Koenig et al., 2010; van Waterschoot et al., 2010). As for the increase of the Papp (A-to-B) values for lopinavir and darunavir in the P-gp KO Caco-2 cells, similar phenomena also have been reported in cells treated with P-gp inhibitors or P-gp knockout mice (Agarwal et al., 2007; Kwan et al., 2009; Holmstock et al., 2010). Among those studies, Agarwal et al. found that the Papp (A-B) of lopinavir increased up to 6 folds in MDCKⅡ-MDR1 treated with P-gp inhibitors. Holmstock et al. also showed that the Papp (A-B) of darunavir increased in Caco-2 cells treated with P-gp inhibitors or in P-gp knockout mice. Many studies demonstrated that the inhibition of P-gp would influence the absorption of different substrates at different extents in Caco-2 cells (Troutman and Thakker, 2003). Missing P-gp at the apical membrane itself or the resultant more significant uptake transporters could influence the uptake of its substrate directly. That might be the reason for the increase of the Papp (A-to-B) values for lopinavir and darunavir in the P-gp KO Caco-2 cells. In addition, BCRP had an impact on nelfinavir and lopinavir transport but not on darunavir, yet there was no active transport of nelfinavir and lopinavir in HEK293-BCRP or MDCKII-Bcrp1 cells (Gupta et al., 2004; Agarwal et al., 2007). *In vitro* studies using chemical inhibitors have also ruled out the interaction of darunavir with BCRP, consistent with our results (Fujimoto et al., 2009; Koenig et al., 2010; Swedrowska et al., 2017). The interaction of darunavir and MRP2 has also been controversial in different studies. A significant suppression of darunavir efflux transport was observed with the MRP2 inhibitor bromsulphthalein (50 µM) in Caco-2 cells, but the cellular accumulation of darunavir was not affected by MRP2 in another study with LS180 cells (Koenig et al., 2010; Swedrowska et al., 2017). Here, it was evident that darunavir was not a substrate of MRP2, as the darunavir was actively efflux transported in the MRP2-knockout Caco^MRP2−/−^ cells (ER 15.8) yet not in MRP2-positive Caco^Pgp−/−BCRP−/−^ cells (ER ∼0.9). In the present study, 5 μM Lopinavir was also not a substrate of MRP2, consistent with many studies (Agarwal et al., 2007; van Waterschoot et al., 2010). However, lopinavir could be transported successfully when the concentration was reduced to 0.5 μM (Agarwal et al., 2007; Janneh et al., 2007). Furthermore, the inhibition experiment showed that the transport activity of MRP2 was inhibited by 5 μM and 10 µM lopinavir (Bierman et al., 2010; Holmstock et al., 2018). This suggests that lopinavir at low level (0.5 μM) could be transported by MRP2, while higher concentration lopinavir would inhibit the activity of MRP2 yet. Therefore, a low concentration was recommended when studying MRP2 mediated lopinavir transport. Remarkably, here we provide the first *in vitro* evidence that MRP2 cannot mediate nelfinavir transport. Compared with other *in vitro* models, more reliable and detailed conclusions could be drawn using the panel of six KO Caco-2 subclones.

Unexpectedly, the efflux ratios of lopinavir and darunavir were significantly increased in some single-KO or double-KO Caco^MRP2−/−BCRP−/−^ cells (lopinavir in Caco^MRP2−/−^ and Caco^MRP2−/−BCRP−/−^, darunavir in Caco^BCRP−/−^ and Caco^MRP2−/−^) compared with WT, but only slightly increased for nelfinavir. The above ER increase was mainly due to a significant decline of Papp (A-to-B). Usually, inhibition of efflux transporters in the apical membrane causes increased A-to-B and decreased B-to-A transport (Mease et al., 2012; Sjöstedt, 2017). On the contrary, all of the groups mentioned above showed a decreased Papp (A-to-B), thus increasing ER. Interestingly, decreased Papp (A-to-B) of digoxin also was found in the BCRP, MRP2, and MRP2/BCRP knock-out cell lines mediated by ZFN (from 0.99 to ~ 0.25 × 10^–6^ cm/s) (Sampson et al., 2015). Further study should be conducted in the future to explore the underlying causes.

## 5 Conclusion

In conclusion, a panel of six rapidly growing Caco-2 subclones with P-gp, BCRP, and MRP2 single and double knockout have been developed successfully by combining CRISPR/Cas9 technology with single cell expansion. We also demonstrated that more reliable and detailed data could be drawn easily with those KO Caco-2 models. These rapidly growing gene-knockout Caco-2 subclones could contribute to efficient *in vitro* drug transport research.

## Data Availability

The datasets presented in this study can be found in online repositories. The names of the repository/repositories and accession number(s) can be found in the article/Supplementary Material.
